# Polymerase I and transcript release factor transgenic mice show impaired function of hematopoietic stem cells

**DOI:** 10.18632/aging.103729

**Published:** 2020-10-21

**Authors:** Lin Bai, Ying Lyu, Guiying Shi, Keya Li, Yiying Huang, Yuanwu Ma, Yu-Sheng Cong, Lianfeng Zhang, Chuan Qin

**Affiliations:** 1 NHC Key Laboratory of Human Disease Comparative Medicine, The Institute of Laboratory Animal Sciences, CAMS&PUMC, Beijing 100021, China; 2 Beijing Engineering Research Center for Experimental Animal Models of Human Critical Diseases, Beijing 100021, China; 3 Institute of Aging Research, Hangzhou Normal University School of Medicine, Hangzhou 310036, China

**Keywords:** PTRF, HSCs, caveolin-1

## Abstract

The age-dependent decline in stem cell function plays a critical role in aging, although the molecular mechanisms remain unclear. PTRF/Cavin-1 is an essential component in the biogenesis and function of caveolae, which regulates cell proliferation, endocytosis, signal transduction and senescence. This study aimed to analyze the role of PTRF in hematopoietic stem cells (HSCs) senescence using PTRF transgenic mice. Flow cytometry was used to detect the frequency of immune cells and hematopoietic stem/progenitor cells (HSCs and HPCs). The results showed than the HSC compartment was significantly expanded in the bone marrow of PTRF transgenic mice compared to age-matched wild-type (WT) mice, and exhibited the senescent phenotype characterized by G1 cell cycle arrest, increased SA-β-Gal activity and high levels of reactive oxygen species (ROS). The PTRF-overexpressing HSCs also showed significantly lower self-renewal and ability to reconstitute hematopoiesis *in vitro* and *in vivo*. Real-time PCR was performed to analyze the expression levels of senescence-related genes. PTRF induced HSCs senescence via the ROS-p38-p16 and caveolin-1-p53-p21 pathways. Furthermore, the PTRF^+^cav-1^-/-^ mice showed similar HSCs function as WT mice, indicating that PTRF induces senescence in HSCs partly through caveolin-1. Thus PTRF impaired HSCs aging partly via caveolin-1.

## INTRODUCTION

Aging is a complex phenomenon that involves the interplay of various factors, such as the functional decline of adult stem cells [1]. Hematopoietic stem cells can self-renew and differentiate into multiple lineages, which is the basis for the maintenance and reconstitution of the entire hematopoietic system. HSCs aging is associated with impaired hematopoiesis in the elderly [2]. In mice for example, several age-dependent changes have been identified in the HSCs, such as increased numbers, decreased homing efficiency, reduced repopulation and self-renewal abilities, and skewed differentiation to the myeloid lineage [3–6]. Studies increasingly show that HSCs aging is regulated by both intrinsic transcriptional factors and extrinsic factors in the niche, and involves the DNA damage repair, cell cycle, telomere maintenance, oxidative stress response and tumor suppression pathways. Despite evidence of impaired HSCs function during aging, the molecular mechanisms are not well understood.

Caveolae are plasma membrane invaginations coated with the scaffolding protein caveolin, and regulate cell growth, endocytosis, mitochondrial function, migration and senescence. The cavin family of proteins is crucial to caveolae formation, stability and dynamics [7–9]. Four cavin proteins have been identified so far, including cavin-1 (PTRF), cavin-2 (SDPR), cavin-3 (SRBC) and cavin-4 (MURC) [10]. Cav-1 null mice have aberrant stem and progenitor cell populations, indicating that Cav-1 regulates stem cell proliferation. In addition, *in vitro* studies using stem cells isolated from these mice suggested that Cav-1 also regulates stem cell differentiation [11, 12]. In our previous study as well, Cav-1 deficient mice exhibited impaired HSCs quiescence, self-renewal and function in response to external factors, although the underlying mechanisms are unknown.

PTRF was first identified as a protein that enhances ribosomal RNA synthesis by dissociating the ternary complex of RNA polymerase I [13]. Hill et al subsequently discovered a regulatory role of PTRF in caveolae biogenesis and function [7]. PTRF-deficient mice lack caveolae, and exhibit dyslipidemia, glucose intolerance and muscular dystrophy [14]. PTRF mutations in humans are also associated with generalized lipodystrophy [15, 16]. We previously showed increased PTRF levels in senescent human fibroblasts compared to young fibroblasts. In addition, overexpression of PTRF in young cells induced features characteristic of senescence, whereas PTRF knockdown extended the replicative lifespan of WI-38 cells [17]. Furthermore, PTRF is down-regulated in breast cancer cell lines and tumors, indicating its potential as a biomarker of breast cancer progression [18]. PTRF transgenic mice are obese and have increased serum levels of ALT and AST, along with greater fat accumulation in the liver compared with WT mice [19]. There is also evidence that PTRF regulates lipid and glucose metabolism in human cells [20].

In this study, we found that PTRF overexpression resulted in HSCs expansion in the bone marrow (BM), which was accompanied by lower repopulation ability and a predominantly myeloid differentiation potential. In addition, the high levels of PTRF in HSCs induced cell cycle arrest at the G1 phase, increased production of reactive oxygen species (ROS) and a higher proportion of SA-β-gal positive cells. Mechanistically, the regulatory effects of PTRF on HSCs function were partly mediated by Cav-1.

## RESULTS

### PTRF expression increased with age and promoted myeloid differentiation

**Figure 1:**
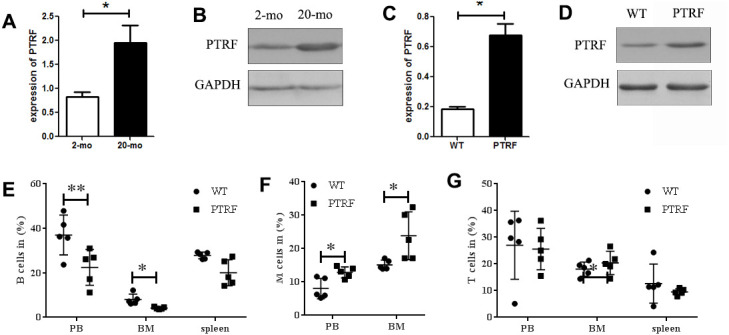
**PTRF expression increased during aging and skewed differentiation potential of HSCs.** (**A**) PTRF mRNA levels in the bone marrow of young (2-mo) and old mice (20-mo), n=3. (**B**) Immunoblot showing PTRF protein expression in the bone marrow (BM) of young and old mice, n=3. (**C**) PTRF mRNA levels in the BM of 2-month old PTRF transgenic and wild-type (WT) mice, n=3. (**D**) Immunoblot showing PTRF protein expression in the BM of young PTRF and WT mice, n=3. (**E**–**G**) The percentage of B cells (**E**), M cells (**F**) and T cells (**G**) in the peripheral blood, BM and spleen of young PTRF transgenic mice. Data represent the mean ± SD of three independent experiments. *, *P* < 0.05; **, *P* < 0.01. Data were normalized against GAPDH expression.

PTRF expression levels increase with age in WI-38 cells and mouse tissues and promote cellular senescence [17, 22]. Consistent with this, PTRF mRNA (Figure 1A) and protein (Figure 1B) levels were significantly higher in the BM cells of 22-month old C57BL/6 mice compared to that of the 2-month old mice. Furthermore, high PTRF levels were also detected in the BM of 2-months old PTRF transgenic mice compared to age-matched WT mice (Figure 1C and 1D). Aged HSCs frequently have a skewed differentiation potential, and produce relatively more myeloid cells compared to lymphoid cells. Compared to the WT mice, the PTRF transgenic mice showed a decreased percentage of B220^+^ B cells in the PB (22.48 ± 8.07 in PTRF vs. 37.04 ± 8.94 in WT) and BM (4.28 ± 0.45 in PTRF vs. 7.99 ± 2.84 in WT) (Figure 1E). Compared to the WT mice, the PTRF transgenic mice showed an increased percentage of CD11b^+^ and Gr-1^+^ M (monocytes and granulocytes) cells (Figure 1F) in the PB(12.88 ± 1.65 in PTRF vs. 8.034 ± 3.06 in WT) and BM(23.86 ± 7.16 in PTRF vs. 15.12 ± 1.44 in WT), although T cells were not affected by the increased PTRF levels (Figure 1G). Taken together, PTRF was upregulated in the BM of aged mice and its ectopic expression increased myeloid differentiation and diminished that to the lymphoid lineage, indicating a crucial role in HSCs aging.

### PTRF overexpression exhausted the HSC pool

**Figure 2:**
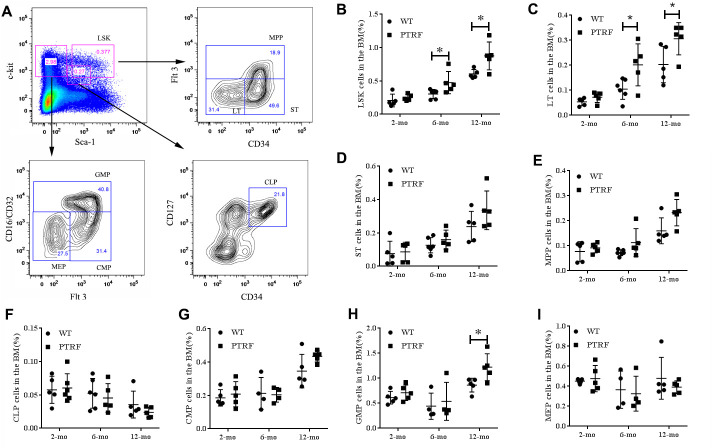
**PTRF overexpression alters the hematopoietic stem/progenitor cell compartments in mice.** (**A**) Representative flow cytometry plots showing HSCs and progenitor populations in the BM. (**B**–**I**) The percentage of LSKs, LT-HSCs, ST-HSCs, MPP, CLP, CMPs, GMPs and MEPs cells in the BM from 2, 6 and 12-month old mice. n=5 mice per group. Data represent the mean ± SD of three independent experiments. *, *P* < 0.05.

To further investigate the role of PTRF in HSC aging, we analyzed the relative proportion of LSKs (Lin^-^Sca1^+^c-kit^+^), LT-HSCs (Lin^-^Sca-1^+^c-Kit^+^CD34^-^Flt-3^-^, long-term HSCs), ST-HSCs (Lin^-^Sca-1^+^c-Kit^+^CD34^+^ Flt-3^-^, short-term HSCs) and MPPs (Lin^-^Sca-1^+^c-Kit^+^Flt-3^+^, multipotent progenitors) in the BM of 2, 6 and 12-month old PTRF and WT mice (Figure 2A). The percentage of LSKs were similar in the young mice, but increased significantly in the 6 and 12-month PTRF mice (1.56-fold, *P*=0.0460 and 1.47-fold, *P*=0.0246) compared to the age-matched WT mice (Figure 2B). The frequency of LT-HSCs also showed an age-dependent increase in the PTRF mice (1.92-fold, *P*=0.0342 and 1.51-fold, *P*=0.0453 at 6 and 12 months respectively) compared to the WT mice (Figure 2C). In contrast, the frequency of ST-HSCs and MPPs were similar in both groups and did not undergo any significant change during aging (Figure 2D and 2E). HSCs differentiate to the common lymphoid progenitors (c-Kit^low^Sca-1^low^Lin^-^, CLPs) and common myeloid progenitors (CD34^+^CD16/CD32^-^, CMPs) in the BM [23, 24]. The latter then differentiate to the more lineage specific granulocyte-macrophage progenitors (CD34^+^CD16/CD32^+^, GMPs) and megakaryocyte-erythroid progenitors (CD34^-^CD16/CD32^-^, MEPs). We observed fewer CLPs (Figure 2F) and an increased proportion of GMPs (Figure 2H) in the PTRF compared to the WT mice, while no significant changes were seen in the percentages of CMPs and MEPs (Figure 2G and 2I). Taken together, PTRF overexpression in the BM correlated with an increased number of aged HSCs, which was most prominent in the LT-HSCs as opposed to the ST-HSCs or MPPs compartments.

### PTRF overexpression impaired HSC function

**Figure 3:**
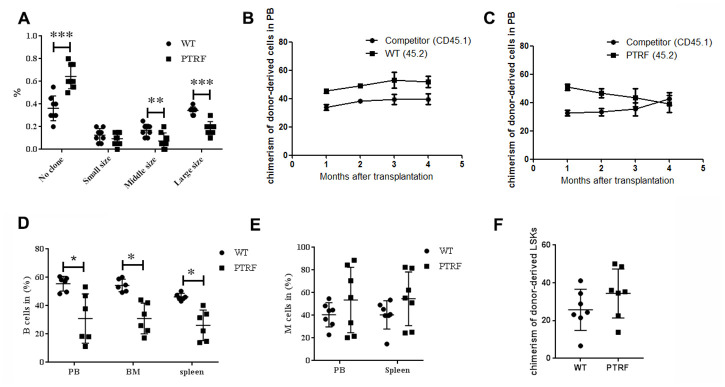
**PTRF impairs HSCs function.** (**A**) Frequency of colonies generated by LT-HSCs from PTRF and WT mice (n=3). (**B**) The percentage of CD45.2+ cells in the PB of irradiated recipient mice 4-, 8-, 12- and 16 weeks after transplantation with WT HSCs. (**C**) The percentage of CD45.2+ cells in the PB of irradiated recipient mice 4-, 8-, 12- and 16 weeks after transplantation with PTRF-overexpressing HSCs. (**D**, **E**) The percentage of donor-derived B (**D**) and M cells (**E**) from PTRF or WT mice in the BM of recipient mice at 16 weeks post-transplantation. (**F**) The percentage of donor-derived LSKs in the BM of recipient mice at 16 weeks post-transplantation. n=9 mice per group. Data represent the mean ± SD. *, P < 0.05.

The clonogenic potential of LT-HSCs isolated from PTRF and WT mice (n=8) were next analyzed by the routine methylcellulose colony assay. The PTRF-overexpressing cells generated fewer and smaller colonies compared to WT LT-HSCs (Figure 3A), which suggested a defect in the self-renewal capacity of the LT-HSCs. This is consistent with previous observations that the repopulation and self-renewal abilities of aged murine HSCs are reduced despite an age-related increase in their number. To further analyze the effect of PTRF on the self-renewal and differentiation capacity of HSCs, we transplanted equal numbers of donor PTRF/WT CD45.2^+^ cells and competitor CD45.1^+^ cells into lethally irradiated mice (CD45.1/CD45.2). The recipient mice showed lower engraftment of the PTRF-derived cells (Figure 3C) compared to that of the WT-derived cells (Figure 3B) at different time points after transplantation. In addition, the percentage of donor-derived B cells was decreased in the PB (30.97 ± 10.87 in PTRF vs. 52.45 ± 3.04 in WT), BM (30.95 ± 17.41 in PTRF vs. 53.45 ± 5.32 in WT) and spleen (26.12 ± 10.80 in PTRF vs. 46.75 ± 0.92 in WT) (Figure 3D) 16 weeks post-transplantation in the mice receiving PTRF-derived cells. The percentage of donor-derived M cells was increased (Figure 3E) 16 weeks post-transplantation in the mice receiving PTRF-derived cells. Compared to the WT mice, the percentage of LSKs was also higher in the mice transplanted with PTRF-derived cells (Figure 3F). Although the above changes did not reach statistical significance, we can conclude from the results that PTRF-overexpressing HSCs failed to reconstitute hematopoiesis in irradiated recipients and had altered differentiation potential.

### PTRF induced HSCs senescence

**Figure 4:**
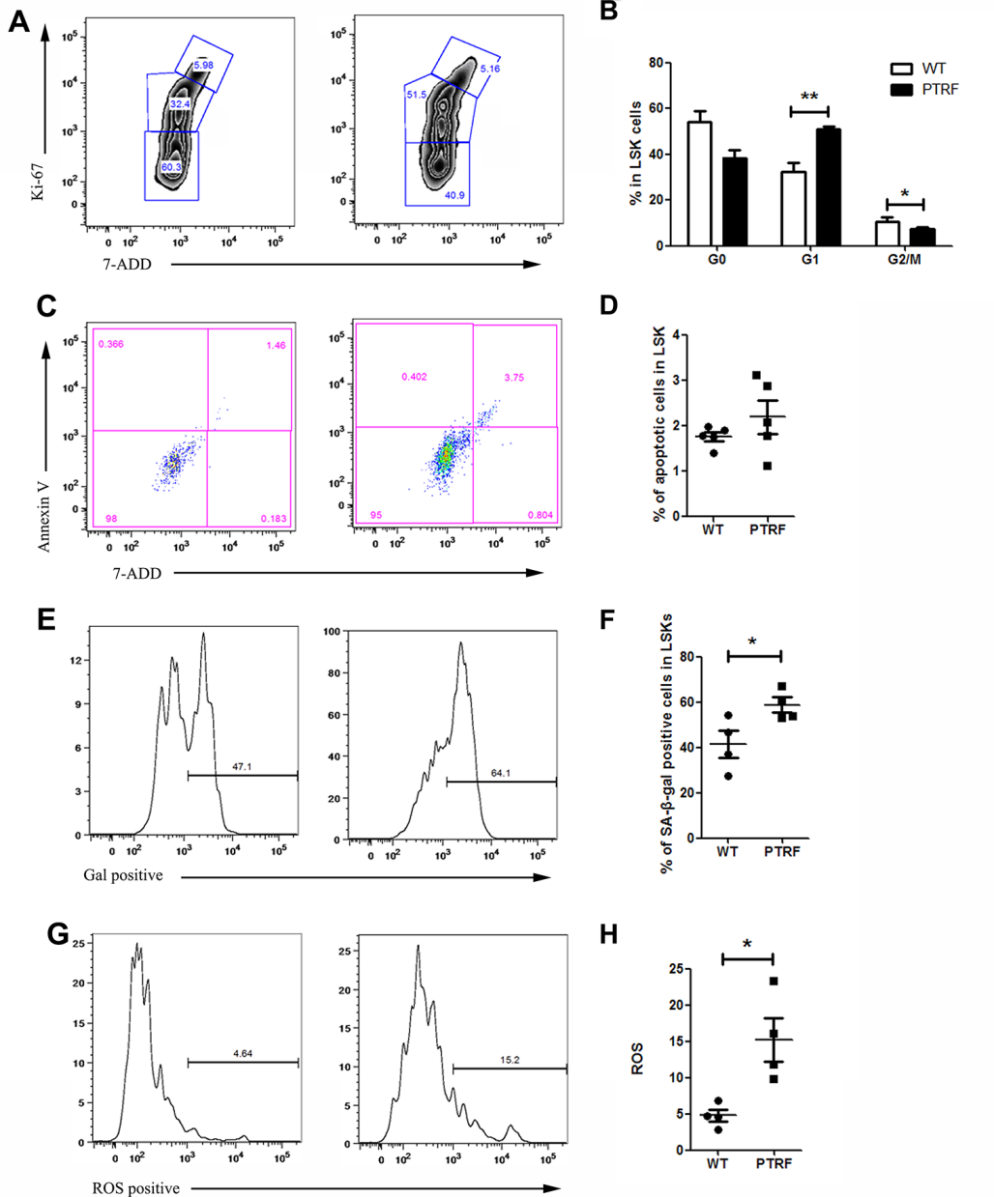
**PTRF overexpression induced cell cycle arrest at G1 phase and accelerated cellular senescence.** (**A**) Flow cytometry plots showing cell cycle distribution of LSKs from PTRF and WT mice. (**B**) The percentage of cells in each phase of the cell cycle. (**C**) Flow cytometry plots showing viable and apoptotic LSKs from PTRF and WT mice. (**D**) The percentage of apoptotic LSK cells. (**E**) Flow cytometry plots showing C_12_FDG levels in the LSKs from PTRF and WT mice. (**F**) The percentage of SA-β-gal-positive LSK cells. n=4 or 5 mice per group. (**G**) Flow cytometry plots showing ROS producing cells stained with the DCFH probe. (**H**) The percentage of ROS-positive LSK cells. Data represent the mean ± SD. *, *P* < 0.05; **, *P* < 0.01.

Since most LT-HSCs in adult mice remain quiescent [26], the effect of PTRF on the frequencies of quiescent or cycling LT-HSCs was also analyzed (Figure 4A). The BM of PTRF-overexpressing mice exhibited an increased percentage of LT-HSCs in the G1 phase (50.97 ± 5.39% in PTRF vs. 32.2 ± 7.93% in WT, *P*=0.0039) with a concomitant decrease in the S–G2/M phase (7.29 ± 1.59% in PTRF vs. 10.62 ± 1.61% in WT, *P*=0.023). However, PTRF had no effect on the number of quiescent (G0) LT-HSCs (38.42 ± 10.16% in PTRF vs. 54.3 ± 11.45% in WT) (Figure 4B). In addition, there were no significant differences in the frequencies of apoptotic HSCs between the PTRF and WT mice (Figure 4C and 4D). Thus, PTRF overexpression induced cell cycle arrest in the HSCs at the G1 phase rather than affect quiescence and apoptosis. Consistent with this, the LSKs from PTRF mice showed higher levels of the fluorescent β-gal substrate C_12_FDG (Figure 4E), and an increase in the percentage of cells with SA-β-gal activity compared to the WT cells (58.8 ± 6.57% in PTRF vs. 41.4 ± 11.7% in WT) (Figure 4F), indicating senescence induction. The loss of quiescence frequently correlates with increased levels of reactive oxygen species (ROS) in the LT-HSCs, which in turn impairs their self-renewal [26]. Consistent with the results so far, PTRF-overexpressing LSKs showed higher levels of ROS compared to that from WT mice (15.26 ± 5.98% in PTRF vs. 4.78 ± 1.65% in WT) (Figure 4G and 4H).

Taken together, PTRF induces a senescent phenotype in the HSCs.

### PTRF activated the senescence pathway in HSCs via Cav-1

**Figure 5:**
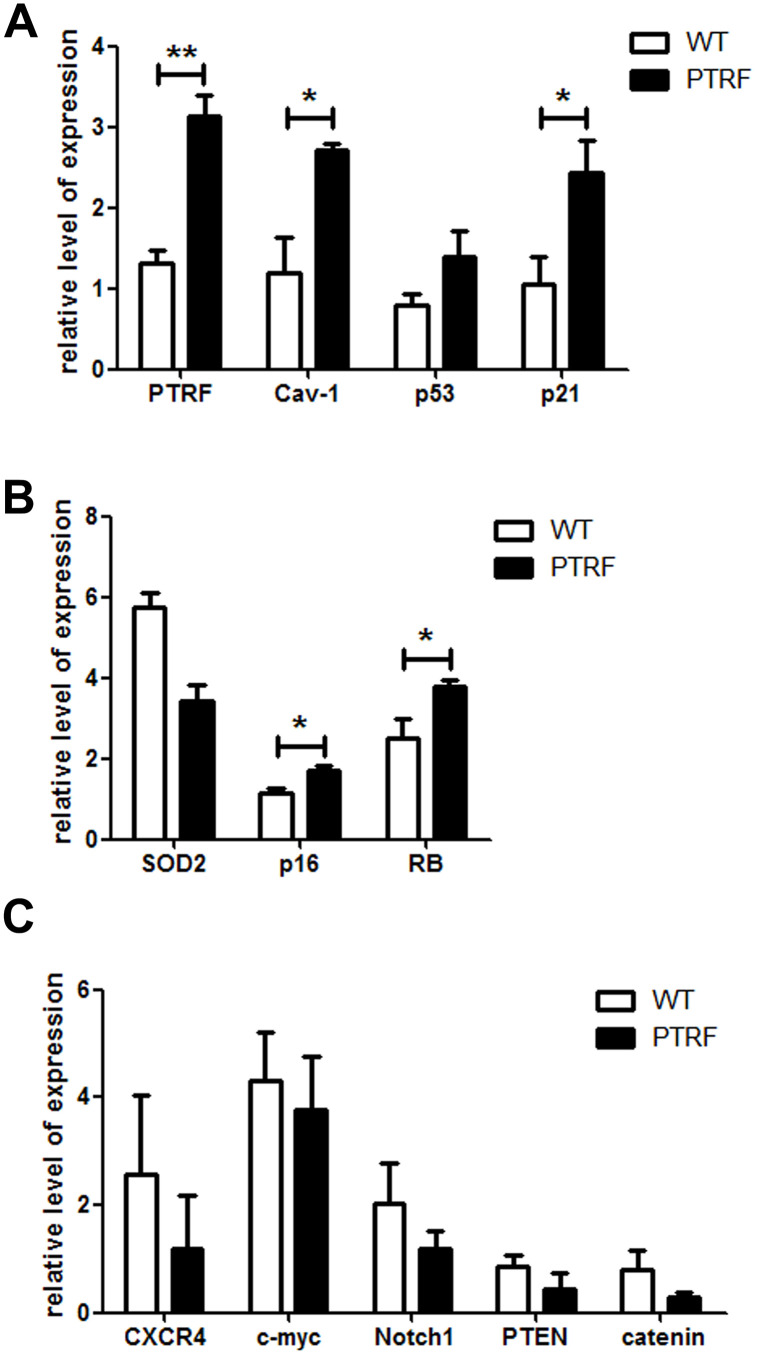
**PTRF altered the expression pattern of senescence-associated genes in LSKs.** (**A**–**C**) RT-PCR analysis of the indicated genes in sorted LSK cells from PTRF and WT mice. Data represent the mean ± SD of three independent experiments. *, *P* < 0.05; **, *P* < 0.01. Data were normalized against GAPDH expression.

**Figure 6:**
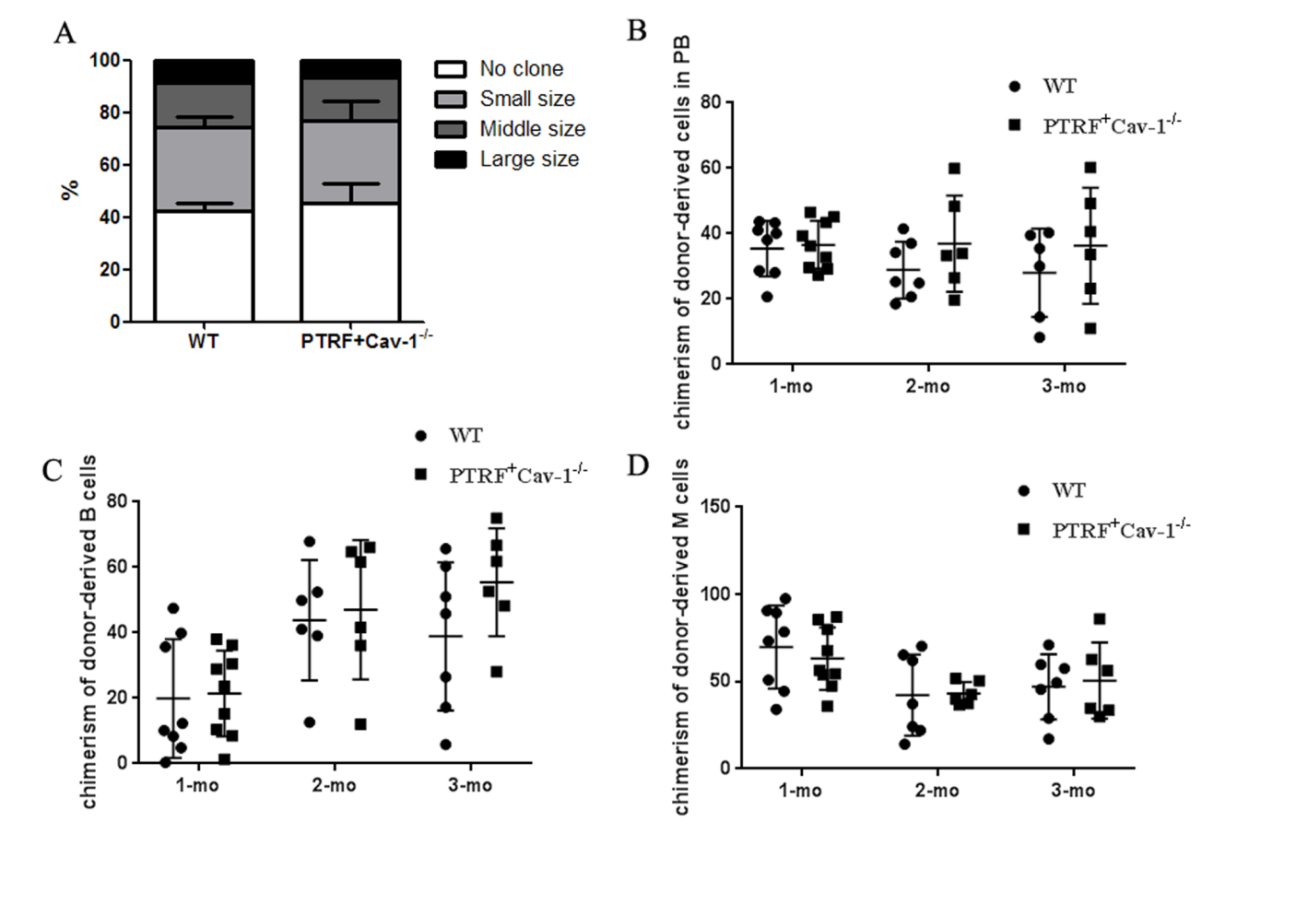
**PTRF impaired HSCs function partly through Cav-1.** (**A**) Number of colonies generated by LT-HSCs from PTRF+Cav-1^-/-^ and WT mice (n=3). (**B**) The percentage of donor-derived cells in the PB of PTRF+Cav-1^-/-^ and WT mice at 4, 8, and 12 weeks post-transplantation. (**C**, **D**) The percentage of donor-derived B (**C**) and M cells (**D**) from PTRF+Cav-1^-/-^ and WT mice in the PB at 4, 8, and 12 weeks post-transplantation. n=9 mice per group. Data represent the mean ± SD.

Cellular senescence and growth arrest are established and maintained by the p53 and p16-pRB pathways. PTRF overexpression in the HSCs significantly upregulated p21, and also induced a slight increase in p53 levels (Figure 5A). p16 and RB were also upregulated in the HSCs of PTRF mice (Figure 5B). However, there were no significant differences in the expression of HSC-related genes between the PTRF and WT mice (Figure 5C). These results indicated that PTRF induced HSC senescence via the p53-p21 and p16-RB pathways. We also detected increased levels of Cav-1 in the PTRF HSCs (Figure 5A), which is significant since PTRF-induced cellular senescence in WI-38 cells is dependent on caveolar targeting and Cav-1 interaction [17]. To further evaluate the role of the Cav-1/p53/p21 pathway in PTRF-induced HSC senescence, we crossed the PTRF transgenic mice with Cav-1 knockout mice to generate PTRF+Cav-1^-/-^ mice. HSCs isolated from the PTRF+Cav-1^-/-^ mice and WT mice showed no difference in the clonogenic capacity both in terms of the number and size of colonies (Figure 6A). Furthermore, competitive transplantation showed no obvious differences in the chimerism of donor-derived B cells and M cells between the PTRF+Cav-1^-/-^ and WT mice (Figure 6B–6D). Taken together, loss of Cav-1 partly compensated the PTRF-induced defects in HSC function, indicating that PTRF initiates the senescence pathway in HSCs via Cav-1.

## DISCUSSION

PTRF is an important factor regulating HSC senescence, as indicated by the increase in cell numbers, impaired self-renewal and hematopoietic reconstitution, and a skewed differential potential of the HSCs isolated from PTRF transgenic mice. Mechanistically, PTRF arrested the LSKs in the G1 phase, increased ROS production and induced senescence via the Cav-1-p53-p21 and p16-RB pathways. Furthermore, the impaired HSCs function in the PTRF transgenic mice was partially abrogated by knocking out Cav-1. Despite an age-dependent increase in the number of HSCs in mice, their repopulating and self-renewing capacities are markedly reduced. The PTRF mice also showed an increased HSCs pool, especially which of LT-HSCs compared to age-matched WT controls. However, *in vitro* clonogenic assays and *in vivo* competitive transplantation showed decreased self-renewal and hematopoietic reconstitution abilities of the PTRF-overexpressing HSCs. Furthermore, the myeloid-directed differentiation potential of the HSCs and a defective B-lymphoid compartment in these mice may have led to HSC exhaustion.

The molecular mechanisms underlying the age-dependent decline in HSC self-renewal capacity are complex, and involve multiple intrinsic and extrinsic factors. The p16-RB and p53-p21 pathways regulate cellular senescence and thus play important roles in the aging process. In fact, p16 levels increase in many human and rodent tissues with aging [27, 28]. In the HSCs, the age-related increase of p16 correlates with decreased self-renewal ability [6, 25]. Consistent with this, p16 knockout enhanced the regenerative potential of HSCs in aging mice [6]. In the p53-deficient mice as well, the number of HSCs increase and their ability to engraft and repopulate the ablated hematopoietic compartment also improves, indicating that the self-renewal capacity of HSCs is affected by p53 as well [29].

PTRF is a key regulator in the biogenesis and function of caveolae, and its functions are associated with Cav-1, which is known to regulate the p53/p21, focal adhesion kinase, epidermal growth factor receptor and small Rho GTPase pathways. Cav-1 overexpression induces premature senescence and activates the p53 pathway [17, 30, 31]. It binds directly to the signaling intermediates via its scaffolding domain. The p53 negative regulator MDM2 has a Cav-1 binding motif, and is sequestered by Cav-1 into caveolar membranes, thereby limiting its interaction with p53. This stabilizes p53 and upregulates the downstream p21, resulting in cell cycle arrest and premature senescence in WI-38 and mouse embryonic fibroblasts [32].

The PTRF induced the number of HSCs increased significantly in the 6 and 12-month PTRF mice. And we previously showed increased PTRF in young human fibroblasts induced features characteristic of senescence. PTRF may impair HSCs and its niche cells functions, which directly or indirectly influence the HSCs function.

PTRF impairs HSC self-renewal and function by inducing senescence via the ROS-p38-p16 and caveolin-1-p53-p21 pathways, which can be partially abrogated by inhibiting Cav-1.

## MATERIALS AND METHODS

### Animals

The PTRF transgenic mice [19] were crossed with the Cav-1^-/-^ strain to generate the PTRF^+^Cav-1^-/-^ mice. The recipient mice were the B6.SJL-Ptprc^a^Pep3^b^/BoyJ (CD45.1) and CD45.1/CD45.2 heterozygous stains. All mice had the C57BL/6J genetic background. The animals were kept in a pathogen-free environment and fed standard diet. All animal experiments were approved by the Animal Care and Use Committees of the Institute of Laboratory Animal Science of Peking Union Medical College.

### Flow cytometry

The BM was flushed out of the tibia and femurs with sterile PBS and homogenized by repeatedly passing through an 18-gauge needle. The spleens and thymi were gently homogenized in a glass homogenizer and the cells were suspended in sterile PBS. Peripheral blood (PB) was incubated with red blood cell lysis buffer (BD Biosciences, San Jose, CA, USA) according to the manufacturer’s instructions and washed with PBS. All cell suspensions were strained through a sterile nylon mesh (75μm) to remove debris and counted. After adjusting the cells to the appropriate density, they were stained with following antibodies for 30 min at 4°C: biotin-conjugated anti-mouse CD4 (RM4-5), CD8a (53–6.7), CD11b (M1/70), B220 (RA3-6B2), TER119 (TER-119) and Gr1 (RB6-8C5), APC-eFluor®780-Streptavidin, PE-Cy7 B220 (RA3-6B2), PE-Cy7 Sca-1 (D7), PE Flt3 (A2F10), FITC B220 (RA3-6B2), FITC CD34 (RAM34), PerCP-Cy5.5 CD127 (A7R34), PE CD16/CD32 (93), PerCP-Cy5.5 CD3e (145-2C11), PerCP-Cy5.5 CD45.2 (104), APC-eFluor®780 CD11b, PE CD45.1 (A20) and APC CD117 (ACK2). All antibodies were purchased from eBioscience (San Diego, CA, USA). The stained cells were acquired on FACS Aria II (Becton Dickson, Franklin Lakes, NJ, USA), and the data were analyzed using FlowJo software (Three Star, Ashland, OR, USA)

### Cell cycle and apoptosis analysis

For cell cycle analysis, total BM cells were first treated with the RBC lysis buffer, and then stained with the stem cell markers (lineage, Sca-1 and c-kit) as described. After fixing and permeabilizing using a pre-formulated buffer (Becton Dickson, 00-5123-43), the cells were stained with FITC-Ki-67 antibody and 7-AAD. To detect apoptosis, total BM cells were stained for stem cell surface markers as above, washed, and then stained with FITC-annexin V antibody and 7-AAD. The cells were acquired on FACS Aria II, and the cell cycle distribution and percentage of apoptotic cells were analyzed using the FlowJo software.

### SA-β-gal staining

SA-β-gal activity was assayed using C_12_FDG (Molecular Probes) according to the manufacturer’s instructions [21]. Briefly, BM cells stained for stem cell markers were incubated with 100nM bafilomycin A1 for 1 h at 37°C to induce lysosomal alkalinization. After washing with PBS, the cells were incubated with 2mM C_12_FDG for 1–2 h at 37°C. The stained cells were analyzed by flow cytometry as described.

### Detection of ROS

BM cells were incubated with DCFH-DA (Beyotime Company, China) at 37°C for 20 min, and the oxidized fluorescent product 2′,7′-dichlorofluorescein (DCFH) was detected by flow cytometry.

### Competitive transplantation

BM cells (2 x 10^6^) from 2-month-old PTRF transgenic, WT and PTRF^+^cav-1^-/-^ mice (donor, CD45.2) were mixed with equal number of BM cells from competitor (CD45.1^+^) mice, and transplanted into lethally irradiated hosts (CD45.1/CD45.2). The animals were sacrificed at 4, 8, 12 and 16 weeks after transplantation, and donor-derived chimerism in the PB and BM were analyzed by flow cytometry to evaluate HSC-mediated reconstitution.

### Clonogenic assays

Suitable cells were plated in 96-well plates at the density of 1 cells/well, with 20 replicates per sample, in a methylcellulose-based medium (R&D, HSC007). After culturing for 14 days, the ensuing colonies were counted under a microscope. Based on the approximate cell load, the colonies were defined as large (>10,000 cells), medium (1,000–10,000) and small (<1,000).

### RT-PCR

Total RNA was extracted using TRIzol reagent (Invitrogen, Carlsbad, CA, USA) according to the manufacturer’s instructions, and treated with DNase I. The cDNA was synthesized using M-MLV Reverse Transcriptase (Promega) and a poly-dT primer. The RT-PCR primers were as follows: Cav-1 5′- GACCCCAAGCATCTCAACGAC-3′ and reverse 5′-GGATCGCAGAAGGTATGGACG-3′ (Tm=62°C, 27 cycles), p21 ^*waf1/cip1*^ forward 5′-TCCAGACATTCAGAGCCACA-3’ and reverse 5’-CGAAGAGACAACGGCACACT-3′ (Tm=60°C, 30 cycles), p53 forward 5′-CATGAACCGCCGACCTATC-3′ and reverse 5′-TCCCGGAACATCTCGAGGC-3′ (Tm=62°C, 35 cycles), p16 forward 5′-CGAACTCTTTCGGTCGTACCC-3′ and reverse 5′-CGAATCTGCACCGTAGTTGAGC-3′ (Tm=62°C, 35 cycles), and GAPDH forward 5′-GAGCGAGACCCCACTAACAT-3′ and reverse 5′-TTCACACCCATCACAAACAT-3′ (Tm=60°C, 25 cycles). The samples were amplified using the SYBR Premix Ex Taq II (TaKaRa Shuzo, Kyoto, Japan) on the ABI StepOne™ detection system (Applied Biosystems, Foster City, CA, USA).

### Western blotting

The cells were lysed with RIPA buffer (50 mM Tris-Cl, pH 8.0, 100 mM NaCl, 0.1% SDS, 0.5% sodium deoxycholate, 1% NP-40) supplemented with protease inhibitor cocktail (Roche). The total protein concentration of the lysates was measured using a BCA kit, and resolved by SDS polyacrylamide gel (15%) electrophoresis. The protein bands were transferred to nitrocellulose (NC) membranes (Millipore) and incubated overnight with anti-PTRF antibody (BD) at 4°C. After probing with HRP-conjugated anti-rabbit secondary antibody, the positive bands were detected using a chemiluminescent reagent (Santa Cruz, USA).

### Statistical analysis

Statistical analysis was performed using Microsoft Excel and GraphPad Prism. Data were represented as mean±standard deviation (SD) and compared by Student’s *t*-test. *P* < 0.05 was considered statistically significant.

### Ethics approval

All mice used in this study were maintained on a C57BL/6 J genetic background and bred in an AAALAC-accredited facility. The Animal Care and Use Committees of the Institute of Laboratory Animal Science of Peking Union Medical College, China approved the animal protocol (ILAS-BL17001 and ILAS-BL17002).
